# Lack of Genetic Differentiation of Five Triatomine Species Belonging to the *Triatoma rubrovaria* Subcomplex (Hemiptera, Reduviidae)

**DOI:** 10.3390/insects16080822

**Published:** 2025-08-08

**Authors:** Amanda R. Caetano, Lucas B. Mosmann, Thaiane Verly, Stephanie Costa, Jader Oliveira, Constança Britto, Márcio G. Pavan

**Affiliations:** 1 Laboratório de Biologia Molecular e Doenças Endêmicas, Instituto Oswaldo Cruz, Fiocruz, Rio de Janeiro 21040-360, RJ, Brazil; amandar.caetano@gmail.com (A.R.C.); thaianeverly1@gmail.com (T.V.); stephaniemartinscosta@gmail.com (S.C.); cbritto@ioc.fiocruz.br (C.B.); 2 Laboratório de Mosquitos Transmissores de Hematozoários, Instituto Oswaldo Cruz, Fiocruz, Rio de Janeiro 21040-360, RJ, Brazil; lucasbonatom@gmail.com; 3 Faculdade de Ciências Farmacêuticas, Universidade Estadual Paulista (UNESP), Araraquara 01049-010, SP, Brazil; jader.oliveira@unesp.br; 4 Department of Entomology, National Museum of Natural History, Smithsonian Institution, Washington, DC 20560, USA

**Keywords:** triatomines, *Triatoma rubrovaria*, phylogenetics, cytochrome b, Chagas disease

## Abstract

The *Triatoma rubrovaria* subcomplex, comprising several triatomine species, plays a significant role in the transmission of Chagas disease in southern Brazil. Despite morphological distinctions among these species, their genetic differentiation remains poorly understood, particularly in sympatric regions. This study investigates the genetic diversity and phylogenetic relationships through DNA sequencing analysis of five sympatric species within the *T. rubrovaria* subcomplex (*T. rubrovaria*, *T. carcavalloi*, *T. klugi*, *T. circummaculata*, and *T. pintodiasi*), using a 542-bp fragment of the mitochondrial cytochrome b (mtCytb) gene. A total of 84 specimens were collected from six municipalities in Rio Grande do Sul, Brazil, and analyzed alongside laboratory-reared specimens and sequences from the GenBank. Bayesian phylogenetic reconstructions, haplotype networks, and population structure analyses revealed a lack of clear genetic differentiation among the five species, with overlapping intra- and interspecific divergences and shared haplotypes. These findings suggest either a single species exhibiting phenotypic plasticity or a group of incipient species with ongoing gene flow. This study highlights the need for a taxonomic revision and suggests that this group could serve as a valuable model for further genomic research to elucidate potential aspects of phenotypic plasticity and/or sympatric speciation in triatomines.

## Simple Summary

Chagas disease is a serious illness caused by the parasite *Trypanosoma cruzi* and transmitted by kissing bugs. Some of these insects, including species of the *Triatoma rubrovaria* group, occur in southern Brazil and may pose a risk of spreading the disease to humans. Scientists have long classified these insects into different species based on their morphological characters, but it is unclear whether they are molecularly distinct. The correct taxonomical assignment of kissing bugs is relevant to correctly determine their bionomic parameters to design purposeful vector control strategies. Here, we analyzed the genetic material of 84 specimens that morphologically belong to five species collected in the wild. Surprisingly, we found that those individuals were molecularly very similar to each other. This suggests that these insects may not be separate species, or that they are still in the early stages of evolving into different groups. Our findings highlight the need for a taxonomical revision of this group and further studies to determine the true diversity of these insects and assess their role in spreading Chagas disease.

## 1. Introduction

In 1909, the Brazilian medical researcher Carlos Justiniano Ribeiro Chagas announced the discovery of a new disease known as Chagas disease, characterizing the symptoms, the protozoan *Trypanosoma cruzi* as the etiological agent, and the triatomines (Hemiptera: Reduviidae) as vectors [1]. One hundred and sixteen years after the discovery, the global burden of *T. cruzi* infections remains, with an estimated 6 to 7 million cases primarily concentrated in 21 Latin American countries [2]. Even though Chagas disease is listed as one of the 30 candidate diseases for elimination by 2030 [3], vector transmission persists in endemic areas where sylvatic native vector species reinvade insecticide-treated dwellings and colonize peridomestic environments [4].

The initiative of Southern Cone countries in 1991 was responsible for a remarkable decline of *T. cruzi* transmission to humans through blood transfusion and virtual elimination of non-native populations of the primary vector *Triatoma infestans* (Klug 1834) [5]. In Brazil, the infestation of domiciles has been sharply reduced since 2008, and the last residual foci of this vector species were recorded in 2014 in Rio Grande do Sul [6]. In this area, however, the elimination of this primary vector has led to an increased occurrence of native species near human dwellings, including *Triatoma sordida* (Stål, 1859) and *Triatoma rubrovaria* (Blanchard, 1843) [7,8].

*Triatoma rubrovaria* is highly competent to transmit *T. cruzi* through its feces [9], but even though it has eclectic blood feeding habits, it is not frequently found with human blood in the gut (1.3–8.0%) [10,11]. This species is currently grouped according to morphological similarities of adults, geographical location and its positioning into a monophyletic subcomplex (*T. rubrovaria* subcomplex), together with other eight species: *Triatoma carcavalloi* (Jurberg, Rocha, and Lent, 1998), *T. circummaculata* (Stål, 1859), *T. klugi* (Carcavallo, Jurberg, Quaresma, and Galvão, 2001), *T. pintodiasi* (Jurberg, Cunha, and Rocha, 2013), *T. oliveirai* (Neiva, Pinto, and Quaresma, 1939), *T. guasayana* (Wygodzinsky and Abalos, 1949), *T. patagonica* (Del Ponte, 1929), and *T. limai* (Del Ponte, 1929) [12,13]. The first five species and *T. rubrovaria* are sympatric in southern Brazil, and adults are distinguishable by the shape and color patterns of the head (ocelli, clypeus, anteclypeus), thorax (anterolateral angles, lateral carinae, submedian carinae, discal tubercles, scutellum, and stridulatory sulcus), abdomen (dorsal, ventral, and posterior views of the female external genitalia), and male genitalia (pygophore, phallosoma, and paramers) [14,15,16,17].

Recent phylogenetic reconstructions based on mitochondrial markers, including a fragment of the most widely used marker in triatomines, the cytochrome b (mtCytb), confirmed the monophyly of the subcomplex, and evidenced three major clusters reciprocally monophyletic, composed of (i) *T. guasayana* occupying the most external cluster, (ii) *T. patagonica* and *T. rubrovaria*, and (iii) a third cluster with four species subdivided in two subclusters, one with *T. pintodiasi* and *T. circummaculata*, and another with *T. carcavalloi* and *T. klugi* [17]. These species, however, were represented by only 2–5 specimens maintained in laboratory conditions. Considering they live in sympatry in southern Brazil (excepting *T. guasayana* and *T. patagonica*) and there are still rare solid examples of sympatric speciation in nature, especially in triatomines [18], we sought to determine whether the morphological and molecular differentiation observed among the five sympatric species is consistent in the field with an increased sampling, thereby confirming the presence of reproductive barriers indicative of speciation.

## 2. Materials and Methods

A total of 84 specimens of the *T. rubrovaria* subcomplex was captured in six municipalities of Rio Grande do Sul, southern Brazil, distanced 70–150 km from each other (Caçapava do Sul, Encruzilhada do Sul, Lavras do Sul, Cachoeira do Sul, Canguçu, and São Jerônimo), in sylvatic areas 50–100 m distant from houses, where sheep and cattle are frequently found in the pasture. Our active search consisted in looking for triatomines under rocks on the ground and in rudimentary walls of overlapping rocks delimiting the lands (Table A1), since these microhabitats are commonly used as shelters by those insect species [7], and capturing them using stainless steel entomological tweezers. The specimens were identified using dichotomous keys [14,16,19]. Unfortunately, immature samples were damaged during sample preparation/transportation, leaving only the head and part of the thorax and legs intact, and thus it was not possible to identify them to the species level. In this case, the taxonomic identification was based on the relative lengths of the proboscis segments and led to the differentiation of samples into two morphogroups. In *T. rubrovaria*, *T. carcavalloi*, and *T. klugi*, the first segment is longer than the second, which in turn is longer than the third (1 > 2 > 3). In contrast, *T. circummaculata* and *T. pintodiasi* have the third segment as the longest, followed by the first and then the second (3 > 1 > 2) [14,16,19]. In addition, laboratory-reared adult specimens of *T. rubrovaria*, *T. circummaculata*, and *T. carcavalloi* were included in the analysis.

One or two legs of each specimen were dissected, and their DNA extracted individually with the QIAamp DNA Mini kit (Qiagen, Hilden, Germany), following the manufacturer’s protocol. Genomic DNA was amplified by PCR using primers (CYTB7432F and CYTB7433R) flanking a 663-bp region of the mitochondrial cytochrome b gene [20]. PCR reactions were carried out in a 50 μL final volume, containing 5 µL DNA (20 to 25 ng), 5 µL 1XGoTaq^®^ Flexi Buffer (Promega, Madison, WI, USA), 1.6 µL 25 mM MgCl2, 0.5 µL GoTaq^®^ Hot Start PCR enzyme (2.5 U), 0.6 µL 100mM dNTPs (Life Technologies, Carlsbad, CA, USA), 1.2 µL each primer at 10 mM, and 33.1 µL ultrapure water. Thermal cycling was carried out in the GeneAmp PCR System 9700 (Life Technologies, Carlsbad, USA), with the following conditions: Hot Start (3 min, 94 °C), followed by 35 cycles of denaturation (30 s, 94 °C), annealing (45 s, 56 °C) and extension (45 s, 72 °C), and a final step (7 min, 72 °C). Both forward and reverse strands were submitted to DNA Sanger sequencing using the BigDye Terminator v3.1 Cycle Sequencing Kit (Applied Biosystems, Carlsbad, CA, USA), following the manufacturer’s protocol, and run on an ABI 3730 XL automated sequencer (Applied Biosystems, Carlsbad, USA) at the PDTIS/FIOCRUZ Genomic Sequencing Platform (Rio de Janeiro, Brazil).

DNA sequence chromatograms of both DNA strands were visually inspected in the SeqMan Lasergene 7.0 (DNASTAR Inc., Madison, WI, USA) for possible background noises or double peaks, and edited to produce a consensus sequence for each sample. Nineteen sequences from five species belonging to the *T. rubrovaria* subcomplex, three sequences of *T. guasayana*, and three of *T. sordida* (which is closely related to *T. guasayana* [12,21]) were retrieved from GenBank https://www.ncbi.nlm.nih.gov/genbank/ (accessed on 10 December 2024) and used in the analyses (Table A1). Unfortunately, we did not include sequences of *T. limai* and *T. oliveirai*, species not collected and whose sequences are not present in GenBank, and *T. patagonica*, whose sequences available in the public database presented very short sizes (<400-bp). Sequences were aligned with ClustalW [22] and pairwise divergences were calculated in the MEGA X program [23], using the Kimura 2-parameter model of nucleotide substitution.

Bayesian phylogenetic trees were reconstructed with the BEAST v2.6.4 package [24]. Trees were sampled from three independent runs every 10,000 generations from 107 MCMC iterations, discarding the 20% first trees. The birth-and-death model of speciation was imposed, and the nucleotide substitution model was selected using the bModelTest package [25]. The convergence of parameters and proper mixing were inspected by checking if the effective sample sizes (ESSs) were sufficiently large (ESSs ≥ 104 for all parameters). Reliability of the recovered clades was assessed through posterior probability (PP) values. A haplotype network was constructed in popART 1.7, using the median-joining model. Clustering analyses were conducted in the R 4.2.3 environment [26]. Principal Component Analysis (PCA) was performed with the adegenet package [27] and plotted with ggplot2 [28]. Hierarchical Bayesian Population Structure analysis (hBAPS) was performed with the rhierbaps [29] and phytools packages [30] and plotted using the ggplot2 package [28]. Divergence plot was generated using the ggplot2 [28], ggpubr [31], and ggrides [32] packages.

## 3. Results

**Figure 1:**
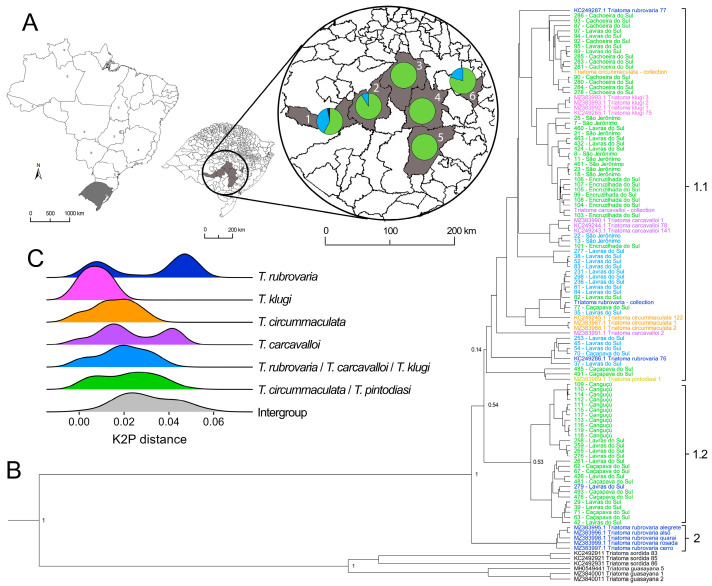
Taxonomic identification, phylogenetics, and molecular distance-based analyses of the *T. rubrovaria* subcomplex. (**A**) Map of Brazil, highlighting the state of Rio Grande do Sul with the municipalities where collections took place indicated in gray. 1—Lavras do Sul; 2—Caçapava do Sul; 3—Cachoeira do Sul; 4—Encruzilhada do Sul; 5—Canguçu; 6—São Jerônimo. The proportion of nymphs identified as belonging to the morphogroups *T. rubrovaria*/*T. carcavalloi*/*T. klugi* (blue) or *T. circummaculata*/*T. pintodiasi* (green), and the *T. rubrovaria* adult (dark blue), in each municipality are shown in pie charts. (**B**) Bayesian phylogenetic reconstruction based on 542-bp of the mtCytb. The locations where samples were collected are listed after sample IDs (Table A1). The numbers near node trees indicate the posterior probability (PP) values. The colors of the tip labels correspond to the taxonomic classification of samples and are consistent with the Figure 1C labels: dark blue—*T. rubrovaria*; pink—*T. klugi*; orange—*T. circummaculata*; purple—*T. carcavalloi*; blue—*T. rubrovaria*/*T. carcavalloi*/*T. klugi* morphogroup; green—*T. circummaculata*/*T. pintodiasi* morphogroup. Moreover, the sequence of *T. pintodiasi* is shown in yellow and the outgroups in black. The brackets on the right represent the hBAPS results as two clusters (1 and 2), of which the first has two subclusters (1.1 and 1.2). (**C**) Frequency of pairwise K2P distances within each group.

Morphological analyses of 83 nymphs (N1–N5) collected in the field allowed the identification of 16 nymphs as a *T. klugi*/*T. rubrovaria*/*T. carcavalloi* morphogroup and 67 nymphs as a *T. circummaculata*/*T. pintodiasi* morphogroup (Figure 1A). A single adult was collected (ID number 279) and identified as *T. rubrovaria* (Table A1). Both morphogroups were found in sympatry in three localities (Caçapava do Sul, Lavras do Sul, and São Jerônimo), while only specimens of the *T. circummaculata*/*T. pintodiasi* morphogroup were found in the other three localities (Cachoeira do Sul, Canguçu, and Encruzilhada do Sul; Figure 1A).

Bayesian phylogenetic reconstruction based on 542-bp of the mtCytb evidenced a clear separation of *T. guasayana* from the other sequences comprising the *T. rubrovaria* subcomplex, evidencing its genetic relatedness to *T. sordida* (Figure 1B). Regarding the other species from the *T. rubrovaria* subcomplex, it is possible to observe two well-supported clusters (Clusters 1.1/1.2 and Cluster 2; PP = 1) that separate five sequences of *T. rubrovaria* generated in da Silva et al. [17] from all other samples used in this study, including those deposited in GenBank. These clusters diverged by an average of 4.7% (3.0–6.2%). The major cluster containing 101 sequences was divided into two weak-supported subclusters (PP = 0.55) with short branches, indicative of low polymorphism between sequences. Cluster 1.1 grouped 73 samples of all five sympatric species and the two morphogroups, while Cluster 1.2 was composed of 28 sequences from three localities (Caçapava do Sul, Canguçu, and Lavras do Sul), of which all but one sample derived from the *T. circummaculata*/*T. pintodiasi* morphogroup.

**Figure A1:**
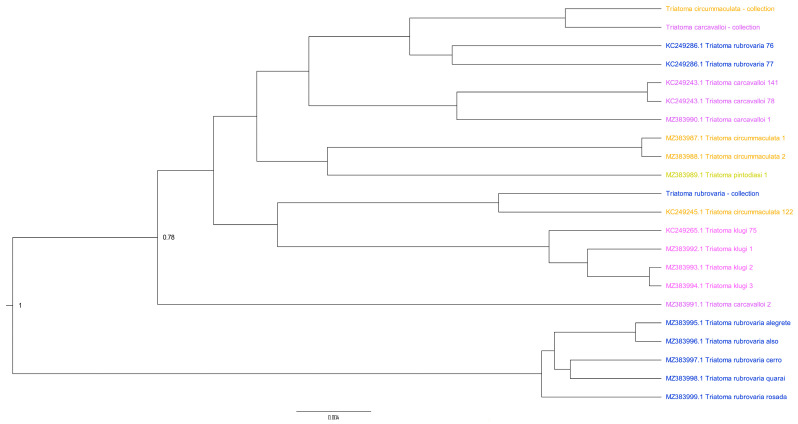
Bayesian phylogenetic reconstruction based on reference sequences from [17]. The numbers near node trees indicate posterior probabilities above 0.7.

Hierarchical BAPS confirmed the separation of the dataset into two subclusters in Cluster 1 (1.1 and 1.2), besides Cluster 2, which was composed of the five sequences of *T. rubrovaria* from da Silva et al. [17]. Pairwise distance analysis (Figure 1C) revealed an overlap of the divergences within and between the morphogroups. The mean intraspecific divergence of sequences for the *T. circummaculata*/*T. pintodiasi* group was 2.2% (0–5.6%), and those within the *T. rubrovaria*/*T. carcavalloi*/*T. klugi* group was 1.8% (0–4.9%), while sequences from the different morphogroups had a mean divergence of 2.6% (0–4.1%). A secondary phylogenetic reconstruction (Figure A1) including only the reference sequences confirmed the separation of the five *T. rubrovaria* sequences from da Silva et al. [17] from all other sequences, and evidenced a paraphyletic pattern across all species, except for *T. klugi*, which was monophyletic, but with low statistical support (PP < 0.6).

**Figure 2:**
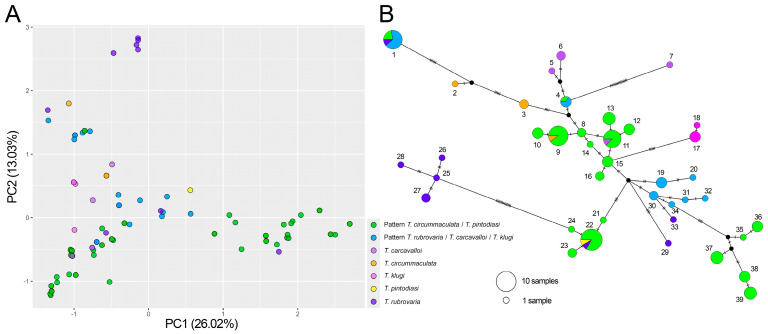
Molecular clustering and structure analyses of the five sympatric species that belong to the *T. rubrovaria* subcomplex. (**A**) Principal component analysis based on the genetic similarity matrix of mtCytb sequences. Color codes for the different morphogroups and species are indicated. (**B**) Haplotype network using the median-joining method. Dashes along the lines indicate the number of mutational steps (>1) that separate the haplotypes. Circle sizes are proportional to the haplotype frequency. In haplotypes shared between species or morphogroups, circles are drawn as pie charts to represent the proportion of samples from each group.

Indeed, PCA did not reveal a clear separation between the morphogroups, of which *T. circummaculata*/*T. pintodiasi* seemed to be the most heterogeneous (Figure 2A). Network analysis (Figure 2B) revealed 39 haplotypes, without a clear separation between the morphogroups. Two haplotypes (1 and 4) were shared with both morphogroups. One haplotype (11) was shared between the *T. circummaculata*/*T. pintodiasi* morphogroup and *T. carcavalloi*, and another haplotype (22) was shared between the *T. circummaculata*/*T. pintodiasi* morphogroup and *T. rubrovaria*.

Phylogenetic analysis, considering only samples morphologically identified at the species level failed to retrieve reciprocally monophyletic groups that could represent *T. rubrovaria*, *T. circummaculata*, and *T. carcavalloi* (Figure A1). Moreover, intra and interspecific divergences also overlap. For instance, intraspecific divergences of *T. carcavalloi* (0–5.2%, mean = 2.3%) and *T. rubrovaria* (0–5.6%, mean = 3.3) were nearly the same as or even greater than the interspecific divergences (1–6.2%, mean = 3.9%).

## 4. Discussion

The mtCytb gene is the most widely used marker for molecular taxonomy in triatomines and has been shown to distinguish closely related species in *Rhodnius* (Stål, 1859) [20,33,34], *Triatoma* (Laporte, 1832) [35,36], and *Panstrongylus* (Berg, 1879) [37] genera. In the case of *T. rubrovaria* subcomplex, our results evidenced a lack of genetic differentiation among five members when increasing field sampling in sympatric areas. The paraphyletic assemblage in the phylogenetic reconstruction, coupled to low genetic divergence and shared haplotypes among different species, indicates that they either represent a single species with phenotypic plasticity or comprise a group of incipient species with few or no barriers for gene flow. Future population genetics studies with fast-evolving markers, such as microsatellites, or with single nucleotide polymorphisms (SNPs) will be essential to clarify this issue.

Phenotypic plasticity enables organisms to express different phenotypes in response to varying environmental conditions, allowing them to adapt without immediate genetic changes [38]. This plasticity can decouple genetic and phenotypic differentiation, as phenotypes may reflect environmental influences rather than underlying genetic divergence [39]. Phenotypic plasticity has already been observed in *Rhodnius nasutus* (Stål, 1859) and *Rhodnius neglectus* (Lent, 1954), in which their body colors matched with the fibers and fronds of their respective palm habitats [5,18,40]. The presence of at least six and two different morphotypes for these species, respectively, supports the idea that they possess genetic traits enabling a range of phenotypes, with natural selection shaping their expression based on environmental factors [18]. There is no evidence, however, of sympatric members of the *T. rubrovaria* subcomplex occupying different niches or with distinct host preferences in sylvatic settings [7]. Microscale ecology studies such as the identification of blood meal sources and microbiota composition could shed light on possible differences on the bionomy of the species.

Phenotypic divergence in closely related species that occur in sympatry may also indicate the occurrence of incipient differentiation that might result in speciation [41]. Genomic studies have revealed that differentiation is restricted to genomic regions of low recombination, usually near centromeres, on early stages of speciation with gene flow [42], and theoretical models predict that sympatric speciation is facilitated when traits under divergent selection influence assortative mating [43]. Members of the *T. rubrovaria* subcomplex represent an excellent model for studying speciation in triatomines. Crossbreeding and genome-wide association studies hold the potential to clarify the mode and pattern of speciation in this group, shedding light on genomic hotspots associated with sympatric speciation. From a public health perspective, determining whether the subcomplex constitutes a single or multiple species is crucial for accurately assessing their vector competence and capacity, and thus mapping areas of risk for Chagas disease transmission.

Intriguingly, five sequences of laboratory-reared *T. rubrovaria* previously published [17], collected in areas 200–460 km away from the areas sampled in this study (Alegrete and Quaraí, Rio Grande do Sul, Brazil), clustered in a well-supported monophyletic clade that diverged 3.0–6.2% (mean = 4.7%) from the other sequences. This percentage of genetic divergence is expected between triatomine sister species [18,20,35,36] and raises the possibility of having allopatric populations of *T. rubrovaria* in Rio Grande do Sul. Unfortunately, it was not possible to include *T. oliveirai* in the analysis, although it is sympatric with the five species studied in this region. However, it is considered rare, having been naturally recorded only twice in burrows of the Brazilian guinea pig, *Cavia aperea* (Erxleben, 1777). [14]. Moreover, the partial preservation of immature specimens constrained the scope of our morphological analysis, particularly for characters located on the abdomen and posterior segments, which are often crucial for accurate taxonomic resolution at the species level. This limitation highlights the importance of proper handling and transportation protocols in entomological surveys. Nevertheless, the reference collection in our laboratory, which includes well-preserved specimens of the identified species, played a crucial role in supporting and confirming the morphological identification based on the available structures. A broader sampling of all species of the *T. rubrovaria* subcomplex would help map their correct geographical distributions and areas of allopatry and sympatry, thus shedding light on historical processes that have shaped the observed genetic diversity.

## Data Availability

All sequences were deposited in GenBank under the accession numbers PV184590-PV184676.

## Abbreviations

The following abbreviations are used in this manuscript:

mtCytb	Mitochondrial Cytochrome b
PP	Posterior Probability
PCA	Principal Component Analysis
hBAPS	Hierarchical Bayesian Analysis of Population Structure
ESS	Effective Sample Size
K2P	Kimura 2-Parameter
MCMC	Markov Chain Monte Carlo

## Appendix A

**Table A1:** Samples and reference sequences used in this study.

Collection Site	Sample ID	Life Stage	Morphogroup/Species	Ecotype	Haplotype	GenBank Accession Number	Reference
Caçapava do Sul	62	Nymph (N5)	*T. circummaculata*/*T. pintodiasi*	Sylvatic	22	PV184590	This study
Caçapava do Sul	63	Nymph (N5)	*T. circummaculata*/*T. pintodiasi*	Sylvatic	22	PV184591	This study
Caçapava do Sul	67	Nymph (N2)	*T. circummaculata*/*T. pintodiasi*	Sylvatic	22	PV184592	This study
Caçapava do Sul	70	Nymph (N3)	*T. rubrovaria*/*T. carcavalloi*/*T. klugi*	Sylvatic	32	PV184593	This study
Caçapava do Sul	71	Nymph (N2)	*T. circummaculata*/*T. pintodiasi*	Sylvatic	22	PV184594	This study
Caçapava do Sul	77	Nymph (N3)	*T. circummaculata*/*T. pintodiasi*	Sylvatic	1	PV184595	This study
Caçapava do Sul	476	Nymph (N2)	*T. circummaculata*/*T. pintodiasi*	Sylvatic	24	PV184596	This study
Caçapava do Sul	481	Nymph (N1)	*T. circummaculata*/*T. pintodiasi*	Sylvatic	15	PV184597	This study
Caçapava do Sul	485	Nymph (N5)	*T. circummaculata*/*T. pintodiasi*	Sylvatic	22	PV184598	This study
Caçapava do Sul	491	Nymph (N5)	*T. circummaculata*/*T. pintodiasi*	Sylvatic	22	PV184599	This study
Caçapava do Sul	493	Nymph (N5)	*T. circummaculata*/*T. pintodiasi*	Sylvatic	22	PV184600	This study
Cachoeira do Sul	87	Nymph (N2)	*T. circummaculata*/*T. pintodiasi*	Sylvatic	9	PV184601	This study
Cachoeira do Sul	90	Nymph (N3)	*T. circummaculata*/*T. pintodiasi*	Sylvatic	10	PV184602	This study
Cachoeira do Sul	92	Nymph (N3)	*T. circummaculata*/*T. pintodiasi*	Sylvatic	9	PV184603	This study
Cachoeira do Sul	93	Nymph (N2)	*T. circummaculata*/*T. pintodiasi*	Sylvatic	10	PV184604	This study
Cachoeira do Sul	278	Nymph (N5)	*T. circummaculata*/*T. pintodiasi*	Sylvatic	9	PV184605	This study
Cachoeira do Sul	280	Nymph (N5)	*T. circummaculata*/*T. pintodiasi*	Sylvatic	10	PV184606	This study
Cachoeira do Sul	281	Nymph (N5)	*T. circummaculata*/*T. pintodiasi*	Sylvatic	9	PV184607	This study
Cachoeira do Sul	283	Nymph (N3)	*T. circummaculata*/*T. pintodiasi*	Sylvatic	9	PV184608	This study
Cachoeira do Sul	284	Nymph (N5)	*T. circummaculata*/*T. pintodiasi*	Sylvatic	9	PV184609	This study
Cachoeira do Sul	285	Nymph (N5)	*T. circummaculata*/*T. pintodiasi*	Sylvatic	9	PV184610	This study
Cachoeira do Sul	286	Nymph (N2)	*T. circummaculata*/*T. pintodiasi*	Sylvatic	14	PV184611	This study
Canguçu	109	Nymph (N2)	*T. circummaculata*/*T. pintodiasi*	Sylvatic	37	PV184612	This study
Canguçu	110	Nymph (N2)	*T. circummaculata*/*T. pintodiasi*	Sylvatic	37	PV184613	This study
Canguçu	111	Nymph (N2)	*T. circummaculata*/*T. pintodiasi*	Sylvatic	38	PV184614	This study
Canguçu	112	Nymph (N2)	*T. circummaculata*/*T. pintodiasi*	Sylvatic	37	PV184615	This study
Canguçu	113	Nymph (N2)	*T. circummaculata*/*T. pintodiasi*	Sylvatic	39	PV184616	This study
Canguçu	114	Nymph (N2)	*T. circummaculata*/*T. pintodiasi*	Sylvatic	37	PV184617	This study
Canguçu	115	Nymph (N2)	*T. circummaculata*/*T. pintodiasi*	Sylvatic	38	PV184618	This study
Canguçu	116	Nymph (N1)	*T. circummaculata*/*T. pintodiasi*	Sylvatic	39	PV184619	This study
Canguçu	117	Nymph (N1)	*T. circummaculata*/*T. pintodiasi*	Sylvatic	38	PV184620	This study
Canguçu	118	Nymph (N2)	*T. circummaculata*/*T. pintodiasi*	Sylvatic	39	PV184621	This study
Canguçu	119	Nymph (N2)	*T. circummaculata*/*T. pintodiasi*	Sylvatic	39	PV184622	This study
Encruzilhada do Sul	99	Nymph (N4)	*T. circummaculata*/*T. pintodiasi*	Sylvatic	11	PV184623	This study
Encruzilhada do Sul	101	Nymph (N4)	*T. circummaculata*/*T. pintodiasi*	Sylvatic	4	PV184624	This study
Encruzilhada do Sul	103	Nymph (N2)	*T. circummaculata*/*T. pintodiasi*	Sylvatic	11	PV184625	This study
Encruzilhada do Sul	104	Nymph (N1)	*T. circummaculata*/*T. pintodiasi*	Sylvatic	11	PV184626	This study
Encruzilhada do Sul	105	Nymph (N2)	*T. circummaculata*/*T. pintodiasi*	Sylvatic	11	PV184627	This study
Encruzilhada do Sul	106	Nymph (N2)	*T. circummaculata*/*T. pintodiasi*	Sylvatic	11	PV184628	This study
Encruzilhada do Sul	107	Nymph (N3)	*T. circummaculata*/*T. pintodiasi*	Sylvatic	11	PV184629	This study
Encruzilhada do Sul	108	Nymph (N3)	*T. circummaculata*/*T. pintodiasi*	Sylvatic	11	PV184630	This study
Lavras do Sul	29	Nymph (N4)	*T. circummaculata*/*T. pintodiasi*	Sylvatic	22	PV184631	This study
Lavras do Sul	35	Nymph (N4)	*T. rubrovaria*/*T. carcavalloi*/*T. klugi*	Sylvatic	1	PV184632	This study
Lavras do Sul	37	Nymph (N4)	*T. circummaculata*/*T. pintodiasi*	Sylvatic	30	PV184633	This study
Lavras do Sul	38	Nymph (N4)	*T. circummaculata*/*T. pintodiasi*	Sylvatic	19	PV184634	This study
Lavras do Sul	39	Nymph (N5)	*T. circummaculata*/*T. pintodiasi*	Sylvatic	22	PV184635	This study
Lavras do Sul	42	Nymph (N5)	*T. circummaculata*/*T. pintodiasi*	Sylvatic	21	PV184636	This study
Lavras do Sul	45	Nymph (N4)	*T. rubrovaria*/*T. carcavalloi*/*T. klugi*	Sylvatic	30	PV184637	This study
Lavras do Sul	52	Nymph (N4)	*T. rubrovaria*/*T. carcavalloi*/*T. klugi*	Sylvatic	19	PV184638	This study
Lavras do Sul	54	Nymph (N4)	*T. rubrovaria*/*T. carcavalloi*/*T. klugi*	Sylvatic	31	PV184639	This study
Lavras do Sul	81	Nymph (N2)	*T. rubrovaria*/*T. carcavalloi*/*T. klugi*	Sylvatic	1	PV184640	This study
Lavras do Sul	82	Nymph (N2)	*T. rubrovaria*/*T. carcavalloi*/*T. klugi*	Sylvatic	1	PV184641	This study
Lavras do Sul	83	Nymph (N5)	*T. rubrovaria*/*T. carcavalloi*/*T. klugi*	Sylvatic	19	PV184642	This study
Lavras do Sul	84	Nymph (N2)	*T. rubrovaria*/*T. carcavalloi*/*T. klugi*	Sylvatic	1	PV184643	This study
Lavras do Sul	89	Nymph (N2)	*T. circummaculata*/*T. pintodiasi*	Sylvatic	9	PV184644	This study
Lavras do Sul	94	Nymph (N3)	*T. circummaculata*/*T. pintodiasi*	Sylvatic	8	PV184645	This study
Lavras do Sul	95	Nymph (N2)	*T. circummaculata*/*T. pintodiasi*	Sylvatic	9	PV184646	This study
Lavras do Sul	97	Nymph (N1)	*T. circummaculata*/*T. pintodiasi*	Sylvatic	8	PV184647	This study
Lavras do Sul	231	Nymph (N5)	*T. rubrovaria*/*T. carcavalloi*/*T. klugi*	Sylvatic	1	PV184648	This study
Lavras do Sul	236	Nymph (N5)	*T. rubrovaria*/*T. carcavalloi*/*T. klugi*	Sylvatic	1	PV184649	This study
Lavras do Sul	253	Nymph (N4)	*T. rubrovaria*/*T. carcavalloi*/*T. klugi*	Sylvatic	34	PV184650	This study
Lavras do Sul	258	Nymph (N5)	*T. circummaculata*/*T. pintodiasi*	Sylvatic	35	PV184651	This study
Lavras do Sul	259	Nymph (N3)	*T. circummaculata*/*T. pintodiasi*	Sylvatic	36	PV184652	This study
Lavras do Sul	261	Nymph (N4)	*T. circummaculata*/*T. pintodiasi*	Sylvatic	22	PV184653	This study
Lavras do Sul	265	Nymph (N5)	*T. circummaculata*/*T. pintodiasi*	Sylvatic	36	PV184654	This study
Lavras do Sul	276	Nymph (N1)	*T. circummaculata*/*T. pintodiasi*	Sylvatic	36	PV184655	This study
Lavras do Sul	277	Nymph (N2)	*T. rubrovaria*/*T. carcavalloi*/*T. klugi*	Sylvatic	20	PV184656	This study
Lavras do Sul	279	Adult (♀)	*Triatoma rubrovaria*	Sylvatic	22	PV184657	This study
Lavras do Sul	298	Nymph (N1)	*T. rubrovaria*/*T. carcavalloi*/*T. klugi*	Sylvatic	1	PV184658	This study
Lavras do Sul	424	Nymph (N5)	*T. circummaculata*/*T. pintodiasi*	Sylvatic	15	PV184659	This study
Lavras do Sul	426	Nymph (N2)	*T. circummaculata*/*T. pintodiasi*	Sylvatic	23	PV184660	This study
Lavras do Sul	432	Nymph (N2)	*T. circummaculata*/*T. pintodiasi*	Sylvatic	15	PV184661	This study
Lavras do Sul	460	Nymph (N2)	*T. circummaculata*/*T. pintodiasi*	Sylvatic	13	PV184662	This study
Lavras do Sul	463	Nymph (N2)	*T. circummaculata*/*T. pintodiasi*	Sylvatic	15	PV184663	This study
São Jerônimo	7	Nymph (N3)	*T. circummaculata*/*T. pintodiasi*	Sylvatic	13	PV184664	This study
São Jerônimo	8	Nymph (N2)	*T. circummaculata*/*T. pintodiasi*	Sylvatic	16	PV184665	This study
São Jerônimo	11	Nymph (N1)	*T. circummaculata*/*T. pintodiasi*	Sylvatic	16	PV184666	This study
São Jerônimo	13	Nymph (N2)	*T. rubrovaria*/*T. carcavalloi*/*T. klugi*	Sylvatic	4	PV184667	This study
São Jerônimo	18	Nymph (N1)	*T. circummaculata*/*T. pintodiasi*	Sylvatic	12	PV184668	This study
São Jerônimo	21	Nymph (N5)	*T. circummaculata*/*T. pintodiasi*	Sylvatic	13	PV184669	This study
São Jerônimo	22	Nymph (N3)	*T. rubrovaria*/*T. carcavalloi*/*T. klugi*	Sylvatic	4	PV184670	This study
São Jerônimo	23	Nymph (N1)	*T. circummaculata*/*T. pintodiasi*	Sylvatic	12	PV184671	This study
São Jerônimo	25	Nymph (N2)	*T. circummaculata*/*T. pintodiasi*	Sylvatic	13	PV184672	This study
São Jerônimo	461	Nymph (N2)	*T. circummaculata*/*T. pintodiasi*	Sylvatic	12	PV184673	This study
Quaraí, RS, Brazil	-	Adult	*Triatoma rubrovaria*	Colony	1	PV184674	This study
São Francisco de Assis, RS, Brazil	-	Adult	*Triatoma carcavalloi*	Colony	11	PV184675	This study
Caçapava do Sul, Brazil	-	Adult	*Triatoma circummaculata*	Colony	9	PV184676	This study
Caçapava do Sul, RS, Brazil	T.rubrovaria76	Adult	*Triatoma rubrovaria*	-	33	KC249286.1	Justi et al. [21]
Quevedos, RS, Brazil	T.rubrovaria77	Adult	*Triatoma rubrovaria*	-	29	KC249287.1	Justi et al. [21]
Quaraí, RS, Brazil	rosada	Adult	*Triatoma rubrovaria*	-	25	MZ383999.1	da Silva et al. [17]
Quaraí, RS, Brazil	quarai	Adult	*Triatoma rubrovaria*	-	26	MZ383998.1	da Silva et al. [17]
Quaraí, RS, Brazil	cerro	Adult	*Triatoma rubrovaria*	-	28	MZ383997.1	da Silva et al. [17]
Alegrete, RS, Brazil	salso	Adult	*Triatoma rubrovaria*	-	27	MZ383996.1	da Silva et al. [17]
Alegrete, RS, Brazil	alegrete	Adult	*Triatoma rubrovaria*	-	27	MZ383995.1	da Silva et al. [17]
São Francisco de Assis, RS, Brazil	T.klugi1	Adult	*Triatoma klugi*	-	17	MZ383992.1	da Silva et al. [17]
São Francisco de Assis, RS, Brazil	T.klugi2	Adult	*Triatoma klugi*	-	17	MZ383993.1	da Silva et al. [17]
São Francisco de Assis, RS, Brazil	T.klugi3	Adult	*Triatoma klugi*	-	17	MZ383994.1	da Silva et al. [17]
Nova Petrópolis, RS, Brazil	T.klugi75	Adult	*Triatoma klugi*	-	18	KC249265.1	Justi et al. [21]
Caçapava do Sul, RS, Brazil	T.carcavalloi1	Adult	*Triatoma carcavalloi*	-	5	MZ383990.1	da Silva et al. [17]
Lavras, RS, Brazil	T.carcavalloi2	Adult	*Triatoma carcavalloi*	-	7	MZ383991.1	da Silva et al. [17]
São Gerônimo, RS, Brazil	T.carcavalloi78	Adult	*Triatoma carcavalloi*	-	6	KC249244.1	Justi et al. [21]
-	T.carcavalloi141	Adult	*Triatoma carcavalloi*	-	6	KC249243.1	Justi et al. [21]
Piratini, RS, Brazil	T.circummaculata122	Adult	*Triatoma circummaculata*	-	2	KC249245.1	Justi et al. [21]
Lavras, RS, Brazil	T.circummaculata1	Adult	*Triatoma circummaculata*	-	3	MZ383987.1	da Silva et al. [17]
Caçapava do Sul, RS, Brazil	T.circummaculata2	Adult	*Triatoma circummaculata*	-	3	MZ383988.1	da Silva et al. [17]
Caçapava do Sul, RS, Brazil	T.pintodiasi1	Adult	*Triatoma pintodiasi*	-	22	MZ383989.1	da Silva et al. [17]
